# Retraction Note: Upregulated long non-coding RNA AGAP2-AS1 represses LATS2 and KLF2 expression through interacting with EZH2 and LSD1 in non-small-cell lung cancer cells

**DOI:** 10.1038/s41419-026-09398-2

**Published:** 2026-11-04

**Authors:** W. Li, M. Sun, C. Zang, P. Ma, J. He, M. Zhang, Z. Huang, Y. Ding, Y. Shu

**Affiliations:** 1 Department of Oncology, First Affiliated Hospital, Nanjing Medical University, Nanjing, People’s Republic of China; 2 Department of Bioinformatics and Computational Biology, UT MD Anderson Cancer Center, Houston, TX USA; 3 Department of Pathology, First Affiliated Hospital, Nanjing Medical University, Nanjing, People’s Republic of China; 4 Jiangsu Key Lab of Cancer Biomarkers, Prevention and Treatment, Collaborative Innovation Center For Cancer Personalized Medicine, School of Public Health, Nanjing Medical University, Nanjing, People’s Republic of China

Retraction Note to: *Cell Death & Disease* https://doi.org/10.1038/cddis.2016.126, published online 19 May 2016

The Editors-in-Chief have retracted this article. Concerns were raised regarding a number of figures, specifically:

- Figure 2d: the lower-left section of the *H1975/si-AGAP2-AS1 1#* panel and the upper-right section of the *H1975/si-AGAP2-AS1 2#* panel appear to overlap;
- Figure 2d and Figure 2C of [1]: the *H1975/si-AGAP2-AS1 1#* panel of Figure 2d and the *H1975/si-LINC01133 1#* panel of Figure 2C of another article [1] from the same author group appear to overlap;
- Figure 3d: sections of multiple panels appear to overlap.

The authors provided some source data on request from the Publisher, but this was insufficient to address the concerns raised. The Editors-in-Chief therefore no longer have confidence in the reliability of the data reported in the article.

W Li, M Sun, J He, M Zhang, and Y Shu did not respond to correspondence from the Publisher about this retraction. The Publisher was unable to contact C Zang, P Ma, Z Huang, and Y Ding.
